# Recurring large deletion in *DRC1* (*CCDC164*) identified as causing primary ciliary dyskinesia in two Asian patients

**DOI:** 10.1002/mgg3.838

**Published:** 2019-07-04

**Authors:** Kozo Morimoto, Minako Hijikata, Maimoona A. Zariwala, Keith Nykamp, Atsushi Inaba, Tz‐Chun Guo, Hiroyuki Yamada, Rebecca Truty, Yuka Sasaki, Ken Ohta, Shoji Kudoh, Margaret W. Leigh, Michael R. Knowles, Naoto Keicho

**Affiliations:** ^1^ Fukujuji Hospital Japan Anti‐Tuberculosis Association Tokyo Japan; ^2^ The Research Institute of Tuberculosis Japan Anti‐Tuberculosis Association Tokyo Japan; ^3^ Department of Pathology and Laboratory Medicine and Marsico Lung Institute University of North Carolina School of Medicine Chapel Hill North Carolina; ^4^ Invitae San Francisco California; ^5^ Japan Anti‐Tuberculosis Association Tokyo Japan; ^6^ Department of Pediatrics and Marsico Lung Institute University of North Carolina School of Medicine Chapel Hill North Carolina; ^7^ Department of Medicine and Marsico Lung Institute University of North Carolina School of Medicine Chapel Hill North Carolina

**Keywords:** Asia, diffuse panbronchiolitis, *DRC1*, primary ciliary dyskinesia, recurrent mutation

## Abstract

**Background:**

Primary ciliary dyskinesia (PCD) is a relatively rare autosomal recessive or X‐linked disorder affecting ciliary function. In the set of causative genes, however, predominant pathogenic variants remain unknown in Asia.

**Method:**

A diagnosis of PCD was made following a modern comprehensive testing including genetic analysis; targeted resequencing for screening variants, and Sanger sequencing for determination of the breakpoints, with an additional review of databases to calculate the deletion frequency. A multiplexed PCR‐based detection method has also been developed.

**Results:**

We ascertained a 50‐year‐old Japanese male who had been diagnosed with diffuse panbronchiolitis (DPB), but refractory to macrolide therapy. We reevaluated the case and identified a large homozygous deletion spanning exons 1 to 4 of the *DRC1* and determined the breakpoints (NM_145038.4: c.1‐3952_540 + 1331del27748‐bp). In the PCD cohort at the University of North Carolina, we found a female PCD patient of Korean descent harboring the same homozygous deletion. From the Invitae testing cohort, we extracted four carriers of the same deletion among 965 Asian individuals, whereas no deletion was found in the 23,951 non‐Asians.

**Conclusion:**

We speculate that the *DRC1* deletion is a recurrent or perhaps founder mutation in Asians. The simple PCR method could be a useful screening tool.

## 1 INTRODUCTION

Primary ciliary dyskinesia (PCD, MIM 244400) is a relatively rare autosomal recessive or X‐linked disorder caused by abnormalities of motile cilia in the respiratory tract and other organs, and of sperm flagella, with an incidence of 10,000–20,000 per birth (Zariwala, Knowles, & Leigh, 2015). Clinical manifestations include chronic rhinosinusitis, recurrent respiratory infections, infertility, and laterality defects. In addition to classical electron microscopy (EM) analysis of ciliary ultrastructure, nasal nitric oxide (nNO) measurements, and genetic analysis are used for diagnosis at well‐equipped PCD centers (Knowles, Zariwala, & Leigh, 2016; Shapiro et al., 2018); whereas, these tests are rarely available in many countries including Japan (European Respiratory Journal 2018 52: PA4427).

In East Asia, diffuse panbronchiolitis (DPB) is well‐known as a multifactorial disease characterized by sino‐pulmonary infection/inflammation with genetic predisposition unique to Asians (Keicho & Hijikata, 2011; Keicho et al., 2000). Although long‐term macrolide therapy dramatically improved the prognosis of the disease in 1980s (Kudoh, Azuma, Yamamoto, Izumi, & Ando, 1998), there still remains DPB patients who are resistant to treatment.

Here, we describe a Japanese PCD patient who had initially been misdiagnosed as refractory DPB. While the ciliary ultrastructure was normal, low levels of nNO prompted us to analyze a panel of PCD genes. A novel homozygous deletion spanning exons 1 to 4 of the *DRC1* (dynein regulatory complex subunit 1, NM_145038.4, [OMIM615288]) was identified (Wirschell et al., 2013; Zariwala et al., 2015). We subsequently reviewed the University of North Carolina (UNC) PCD cohort and found the same homozygous deletion in a patient of Korean origin. Identification of this multi‐exon deletion in several individuals of Asian descent strongly suggests that it is recurrent and possibly a founder mutation in the Asian population. A PCR‐based screening method for this deletion is also proposed here as a useful tool to detect this deletion.

## 2 MATERIAL AND METHODS

A signed and informed consent was obtained from all the participants in this study. All protocols involving human studies were approved by the University of North Carolina Medical School Review Board. This study was approved by each institutional review board (Institutional review board number 16,024 at Fukujuji Hospital, Japan Anti‐Tuberculosis Association (JATA), RIT/IRB 28–20 at the Research Institute of Tuberculosis, JATA, and 20,161,796 at Invitae Corporation). Detailed information about the patients and the diagnostic procedures including genetic analysis are documented in Supporting Information: Briefly, based on prior observation of normal EM findings of nasal cilia from the first Japanese case, his genomic DNA was subjected to targeted resequencing analysis of four genes that are known to cause PCD without ultrastructural changes of cilia. The PCR primers for resequencing are shown in Table S1. Breakpoints of the large deletion of *DRC1* were further determined in the first case by Sanger sequencing and confirmed in the second case at UNC. Multiplexed PCR‐based method was also developed to screen the deletion*.* The frequency of the *DRC*1 deletion was assessed in a total of 24,916 individuals (49,832 total alleles) who had been tested for PCD and non‐PCD phenotypes at Invitae Corporation; ethnicity data for these individuals was self‐reported (see Supporting Information).

## 3 RESULTS

### 3.1 Clinical features of the first case and identification of a novel *DRC1* mutation

**Figure 1:**
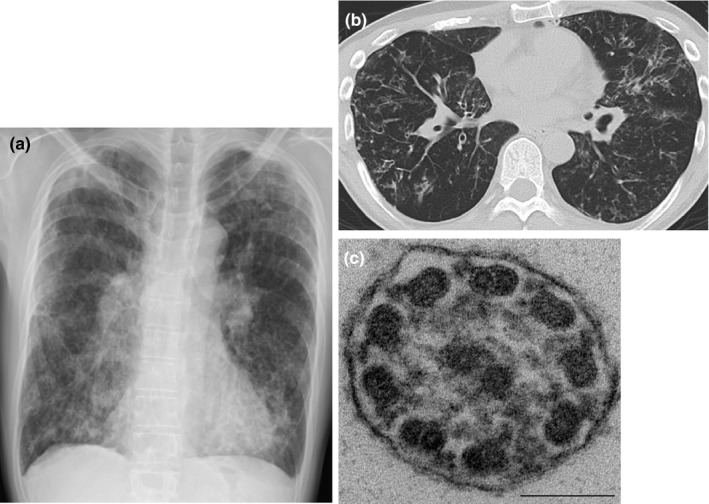
Radiological and electron microscopy findings of case 1 of primary ciliary dyskinesia. (a) Chest X‐ray showing nodular shadows with tramlines and consolidation predominantlyin the lower lung field. (b) Chest CT scans showing small nodules and bronchiectasis in the both lung field. (c) Transmission electron micrograph showing of a ciliary cross section showing normal 9 + 2 microtubular arrangement and presence of both inner and outer dynein arms. Bar = 100 nm

A 50‐year‐old male (case 1) was referred to Fukujuji Hospital because his productive cough and dyspnea had worsened over a few months. The patient was diagnosed with DPB in his late 40 s at a nearby hospital, based on clinical findings, including chronic rhinosinusitis, bilateral centrilobular lesions indicating diffuse bronchiolitis, and obstructive impairment in the pulmonary function test. He was a never‐smoker and did not have any history of rheumatoid arthritis or Sjögren's syndrome. There was no known consanguinity. No situs abnormalities were identified. He had borderline hypoxemia (oxygen saturation 91%) under 0.5 L/min oxygen supplementation. Physical examination revealed coarse crackles in the lung fields. Chest X‐ray showed diffuse granular and reticular shadows with hyperinflation in both lungs. Chest CT scanning showed diffuse bronchiectasis, mainly in the middle lobe (Figure 1). Sinus CT scans were indicative of chronic sinusitis. In most patients with DPB, treatment with clarithromycin leads to dramatic improvement. However, this case was refractory to this treatment. We further noticed that he had a history of fertility treatment, and suspected PCD. We attempted comprehensive tests for diagnosis of PCD, following the current guidelines: EM findings showed a slightly decreased number of inner dynein arms of the cilia but within normal limit (Figure 1), whereas nNO level was low (5.27 nl/min).

**Figure 2:**
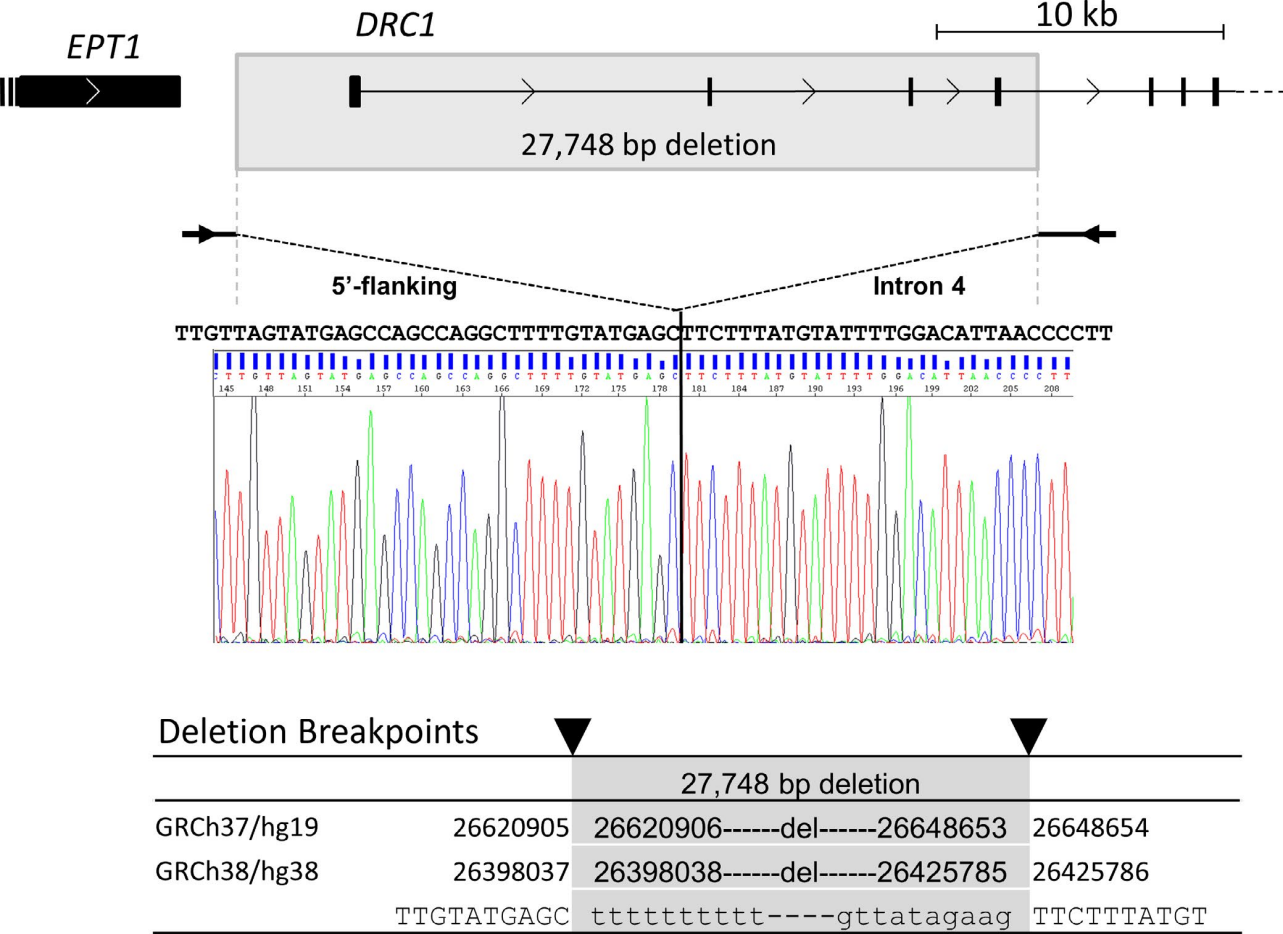
Breakpoints of the deletion in *DRC1* determined in case 1. A genomic region containing the deletion was amplified by PCR and directly sequenced as described in Supporting Information, and the deletion breakpoints were determined

By genetic approach, we focused on sequencing PCD‐causative genes that do not affect ciliary ultrastructure (*DNAH11, DRC1, CCDC65, GAS8, and HYDIN*), which ultimately revealed that zero reads mapped to exons 1 to 4 of *DRC1* suggesting presence of a homozygous deletion encompassing these exons (Figure S1a). The genomic region encompassing the deletion was amplified by PCR (Figure S1b), and direct Sanger sequencing of the PCR product revealed a 27,748 bp deletion with breakpoints shown in Figure 2 (NM_145038.4: c.1‐3952_540 + 1331del27748‐bp). We sebsequently excluded other genetic causes of PCD (*DNAH5, DNAI1, CCDC39, CCDC40, C21orf59, DNAAF1, DNAAF2, DNAAF3, DNAAF4, DNAAF5, LRRC6, SPAG1, ZMYND10, CCNO, RSPH1, RSPH3, RSPH9, RSPH4A, ARMC4, CCDC103, CCDC151, DNAI2, DNAL1, NME8, CCDC114, PIH1D3, DNAJB13, TTC25*).

### 3.2 Discovery of the second case of the same *DRC1* deletion in PCD cohort at UNC

Since our first patient carried a novel, homozygous gross deletion in *DRC1*, we reviewed similar cases in the UNC cohort (Supporting Information) with the consideration that this deletion may be recurrent in the Asian population.

A 5‐year‐old girl (case 2) of Korean descent with bronchiectasis was referred to UNC for evaluation for possible PCD. She was born in South Korea but was adopted by a family in the United States at 13 months of age. She had daily, year‐round wet cough and daily, year‐round nasal congestion and drainage since infancy. She had no laterality defect. She had a chest CT scan at 4.5 years of age that showed bronchiectasis in the right middle lobe, the right lower lobe, and lingula. Evaluation for primary ciliary dyskinesia included low nNO level (8 nl/min), and normal ciliary ultrastructure on EM.

Based on the positions of breakpoints identified in case 1, the genomic region containing the deletion of *DRC1* in case 2 was amplified by PCR and sequenced to confirm the deleted region and breakpoints (Figure S2). A multiplexed PCR‐based method showed the amplification of a single 515 bp fragment from both cases 1 and 2, which indicated the homozygosity of the deletion allele; control genomic DNA show three fragments from the wild type allele (Figure S3).

### 3.3 Ethnicity of the individuals with the large *DRC1* deletion in genetic test panels at Invitae

Here, we have identified two individuals with PCD of Asian descent who are homozygous for the *DRC1* exon 1–4 deletion (cases 1 and 2). In the Invitae database, an additional four individuals were found to be heterozygous for the *DRC1* large deletion. Thus, the overall minor allele frequency (MAF) of the deletion in the Invitae cohort was calculated to be 0.012% (6/49,832). When including only those individuals who self‐reported as Asian (965 individuals), the ethnic specific MAF was calculated to be 0.31% (6/1,930; 95% CI using Wilson method: 0.14%–0.68%). Importantly, the MAF in Asians was significantly higher than other self‐reported ethnicities, i.e. white or Caucasian 95% CI MAF: 0%–0.025%.

## 4 DISCUSSION

In the present study, we report a novel 28‐kb deletion in *DRC1,* a PCD causative gene, carried by Asian patients. Case 1 was initially diagnosed as DPB with respiratory failure, and refractory against macrolide treatment. Surprisingly, nNO levels were low despite no detectable ultrastructural abnormalities in cilia, and a large homozygous deletion in *DRC1* was identified by targeted resequencing approach. A unique group of genes causative for PCD and encoding subunit proteins involving nexin links and the dynein regulatory complex (N‐DRC) have a peculiar characteristic of normal EM findings except for invisible nexin links (Shapiro & Leigh, 2017). In addition, *DRC1* mutations, including cases 1 and 2 reported here, have not been reported to cause laterality defects (Knowles et al., 2016). This is consistent with mutations in other N‐DRC related genes *CCDC65* and *GAS8*. Importantly, if ciliary ultrastructural EM were the sole diagnostic method used for the individuals in this report, they would have been misdiagnosed with refractory DPB since macrolide treatment is known to fail for patients diagnosed with PCD. It is thus essential to evaluate individuals suspected to have PCD comprehensively based on the current guidelines, including appropriate genetic testing.

The same homozygous gross deletion of *DRC1* was observed in two cases. The case 2 had respiratory symptoms within one day after birth. Consistent with earlier reports, *DRC1* mutations may often lead to early onset of the disease (Wirschell et al., 2013). Considering the diagnostic delay in case 1, awareness of PCD might be low among health care providers in Japan and other countries; further education in these countries will be important for early diagnosis and timely treatment of patients with PCD. Establishment of a standard diagnostic process in these countries is also absolutely essential.

The prevalence of *DRC1*‐related PCD is estimated to be <1% in Western literature reports (Zariwala et al., 2015). Importantly, all individuals with the *DRC1* exon 1–4 deletion observed in this study are of Asian descent. In addition, a nearly identical deletion (Chr2:26398037–26425785, esv2657536) has been reported in Japanese (JPT) individual NA19080 of the 1,000 genomes database (http://www.1000genomes.org/). As a result, we speculate that this variant is a founder mutation in the Asian population, although analyses of a large number of definitive cases (Takeuchi et al., 2018) and haplotype analysis on a large dataset will be required to demonstrate this conclusively. Considering ethnicity of the two homozygotes reported herein, it is likely that the *DRC1* deletion is highly recurrent in East Asian populations, particularly in Koreans and Japanese with a similar genetic background based on HLA typing results(Keicho & Hijikata, 2011; Keicho et al., 2000), although future research into the ethnic specific population frequency of this deletion is needed.

In summary, we found two PCD cases homozygous for a gross deletion in *DRC1:* a Japanese male and a Korean female. In addition, four individuals of Asian descent were found to be carriers. Case 1 had been treated as a refractory case of DPB for years. The correct diagnosis of PCD caused by the *DRC1* deletion was made by the methods including nNO and genetic analysis. With our research presented here, we show that the *DRC1* deletion was detected only in Asians. Together with our two cases, we speculate that this is a founder mutation in Asians. Given the likely recurrent nature of the *DRC1* deletion, our screening PCR method could be a useful approach for Asian patients suspected of PCD and refractory DPB.

## CONFLICT OF INTEREST

Rebecca Truty and Keith Nykamp are employees and stockholder of Invitae. Maimoona A. Zariwala and Margaret W. Leigh received research grants from Parison Science and Vertex Pharmaceuticals outside of this study.
