# Supplementary material for: Recurring large deletion in DRC1 (CCDC164) identified as causing primary ciliary dyskinesia in two Asian patients
Source: Mol Genet Genomic Med. 2019 Jul 4;7(8):e838. doi: 10.1002/mgg3.838 (PMC6687623; doi:10.1002/mgg3.838)
Supplement: Supplementary file 2 [file MGG3-7-e838-s002.docx]

**Recurring large deletion in *DRC1* (*CCDC164*) identified as causing primary ciliary dyskinesia in two Asian patients**

**Supporting information**

**Supplementary Methods**

***Patients and diagnostic procedure***

The initial patient (case 1) was a 50-year-old Japanese man who was clinically assessed at Fukujuji Hospital, Japan Anti-Tuberculosis Association (JATA), Tokyo, Japan. Laboratory tests were performed at the Research Institute of Tuberculosis (RIT), JATA, Tokyo. The second patient (case 2) found in the PCD cohort by UNC was a Korean girl born in South Korea and living in the U.S. The diagnosis and evaluation of case 2 were performed at UNC. Know how of nNO and EM analysis were transferred from UNC toward the forthcoming opening of PCD clinic in Tokyo: nNO was collected using the resister according to the guideline-recommended method, and measured by the chemiluminescence method with Sievers 280i NOA (Sievers, Boulder, CO). EM analysis has also followed the standard operation procedure in UNC and evaluated by two Japanese investigators and one UNC staff member (K.M, N.K, and M.K).

***Genetics analysis of case 1***

Based on prior information about normal EM findings of nasal cilia from the patient, all exons of *DNAH11* (dynein axonemal heavy chain 11)(Bartoloni et al., 2002), *DRC1* (*CCDC164*), *CCDC65* (coiled-coil domain containing 65, *DRC2*)(Austin-Tse et al., 2013; Horani et al., 2013) and *GAS8* (growth arrest specific 8, *DRC4*)(Olbrich et al., 2015) were amplified from 40 ng of genomic DNA extracted from the whole blood using 62 primer pairs by multiplexed PCR or by single PCR (**Table S1**). Tks Gflex DNA polymerase (TaKaRa, Shiga, Japan) was used as recommended by the manufacturer with final concentration of 0.25 μM for each primer, and the PCR condition was 94^o^C for 1 min, 2 cycles of 98^o^C 10 sec, 70^o^C 5 min, 2 cycles of 98^o^C 10 sec, 68^o^C 5 min, followed by 40 cycles of 98^o^C 10 sec, 60^o^C 15 sec and 68^o^C for 5 min. Approximately equal molecular amounts of PCR products were collected, mixed and purified using QIAquick PCR purification kit (QIAGEN, Hilden, Germany). One hundred ng of the purified product was subjected to library preparation using QIAseq FX DNA Library Kit (QIAGEN). The library with appropriate size distribution was selected using AMPure XP beads (Beckman Coulter, CA, USA), and 2 ×150 bp paired-end sequencing run was performed using MiSeq Reagent Kit Nano v2 (300 Cycle) and MiSeq System (Illumina, CA, USA). The sequence reads were trimmed, aligned to the human genome (hg38), and variants were called and annotated using CLC Genomics Workbench version 11.0 (QIAGEN).

The genomic region encompassing the large deletion in *DRC1* was amplified with primers 5´- CAGTGGACTAAGGTAGGTGCTTGG -3´ located in *EPT1*-*DRC1* intergenic region and 5´- ACCAAATGGCTTTGACAAGGGTCA -3´ in intron 4 of *DRC1* as described above, with PCR condition of 94^o^C for 1 min followed by 35 cycles of 98^o^C 10 sec, 60^o^C 15 sec and 68^o^C for 1 min 30 sec. The PCR product was purified with QIAquick PCR purification kit (QIAGEN), and sequenced bidirectionally with primers 5´- GGCAGTCAGTCCTACATATCAAGG -3´ or 5´- TAAGAAGGCAATATACGGAATAGG -3´, using BigDye terminator v3.1 cycle sequencing kit and SeqStudio Genetic Analyzer (Thermo Fisher Scientific, MA, USA).

***Genetics analysis of case 2 in UNC***

The initial whole exome sequencing followed by the analysis of the genes known to be associated with PCD did not yield biallelic pathogenic variants in the patient. To study if the copy number variant (CNV) played a role in the pathogenicity, a research testing of 31 genes associated with PCD was carried out at Invitae, San Francisco, CA, which revealed a large homozygous deletion spanning exons 1 to 4 in *DRC1*. Based on the positions of breakpoints determined in case 1, amplification of the genomic region encompassing the large deletion was tried by PCR and sequenced by the Sanger method.

***Multiplexed PCR-based method to screen the large deletion in DRC1***

Multiplexed PCR-based method was also developed to screen the large deletion in *DRC1* using M13 tagged sense primer from 5’UTR (5’-tcagggatgaggaactcgac-3’) and antisense primer from intron 4 (5’-tttctgcacattggatttgg-3’). The primers from outside the deletion region detected a 515 bp fragment in individuals carrying deletion. *DRC1* primer pairs from flanking regions of exon 2 (600-bp), exon 3 (376-bp) and exon 4 (849-bp) were used as an internal control for amplification as well as to decipher homozygosity versus heterozygosity of the large deletion. Primer sequences are as follows: Exon 2 sense (5’- gctattttggccatgctgat -3’) and exon 2 antisense (5’- tggaaggcccaaattaggta -3’). Exon 3 sense (5’- ggtagttgctgcttcctgct -3’) and exon 3 antisense (5’- agggagggagggagaagtta -3’). Exon 4 sense (5’- tgagggagttatgcaacttgtc -3’) and exon 4 antisense (5’- ggagtgaaccacctcatcca -3’). PCR reaction in a total volume of 20μl was carried out using final concentration of 1X PCR buffer, 1.5mM MgCl_2_, 0.4 μM each primer, 100 μM dNTP, 0.05 units of Taq polymerase from Sigma-Aldrich (St. Louis, MO) and 20-200 ng genomic DNA, with PCR condition of 94^o^C for 5 min followed by 35 cycles of 94^o^C 30 sec, 60^o^C 45 sec, 72^o^C for 45 sec, and final extension of 72^o^C for 10 min.

***Ethnicity data of PCD and non-PCD genetic panels at Invitae corporation***

A total of 24,916 individuals were tested for the DRC1 gene at Invitae. Information on self-reported ethnicity data is as follows: Asian, mixed (Asian plus another ancestry), 51; Asian, 965; Ashkenazi Jewish, 1009; Other (self-reported as mixed ancestry or something very specific), 1291; Black/African-American, 1374; Hispanic, 1817; Unknown, 3154; White/Caucasian, 15255.

**Supplementary References**

Austin-Tse, C., Halbritter, J., Zariwala, M. A., Gilberti, R. M., Gee, H. Y., Hellman, N., . . . Hildebrandt, F. (2013). Zebrafish Ciliopathy Screen Plus Human Mutational Analysis Identifies C21orf59 and CCDC65 Defects as Causing Primary Ciliary Dyskinesia. *Am J Hum Genet, 93*(4), 672-686. doi:10.1016/j.ajhg.2013.08.015

Bartoloni, L., Blouin, J. L., Pan, Y., Gehrig, C., Maiti, A. K., Scamuffa, N., . . . Antonarakis, S. E. (2002). Mutations in the DNAH11 (axonemal heavy chain dynein type 11) gene cause one form of situs inversus totalis and most likely primary ciliary dyskinesia. *Proc Natl Acad Sci U S A, 99*(16), 10282-10286. doi:10.1073/pnas.152337699

Horani, A., Brody, S. L., Ferkol, T. W., Shoseyov, D., Wasserman, M. G., Ta-shma, A., . . . Kerem, E. (2013). CCDC65 mutation causes primary ciliary dyskinesia with normal ultrastructure and hyperkinetic cilia. *PLoS One, 8*(8), e72299. doi:10.1371/journal.pone.0072299

Olbrich, H., Cremers, C., Loges, N. T., Werner, C., Nielsen, K. G., Marthin, J. K., . . . Omran, H. (2015). Loss-of-Function GAS8 Mutations Cause Primary Ciliary Dyskinesia and Disrupt the Nexin-Dynein Regulatory Complex. *Am J Hum Genet, 97*(4), 546-554. doi:10.1016/j.ajhg.2015.08.012

**Figure S1**

**PCR amplification of *DRC1* exons (A) and genomic region containing the deletion (B) in case 1**

**
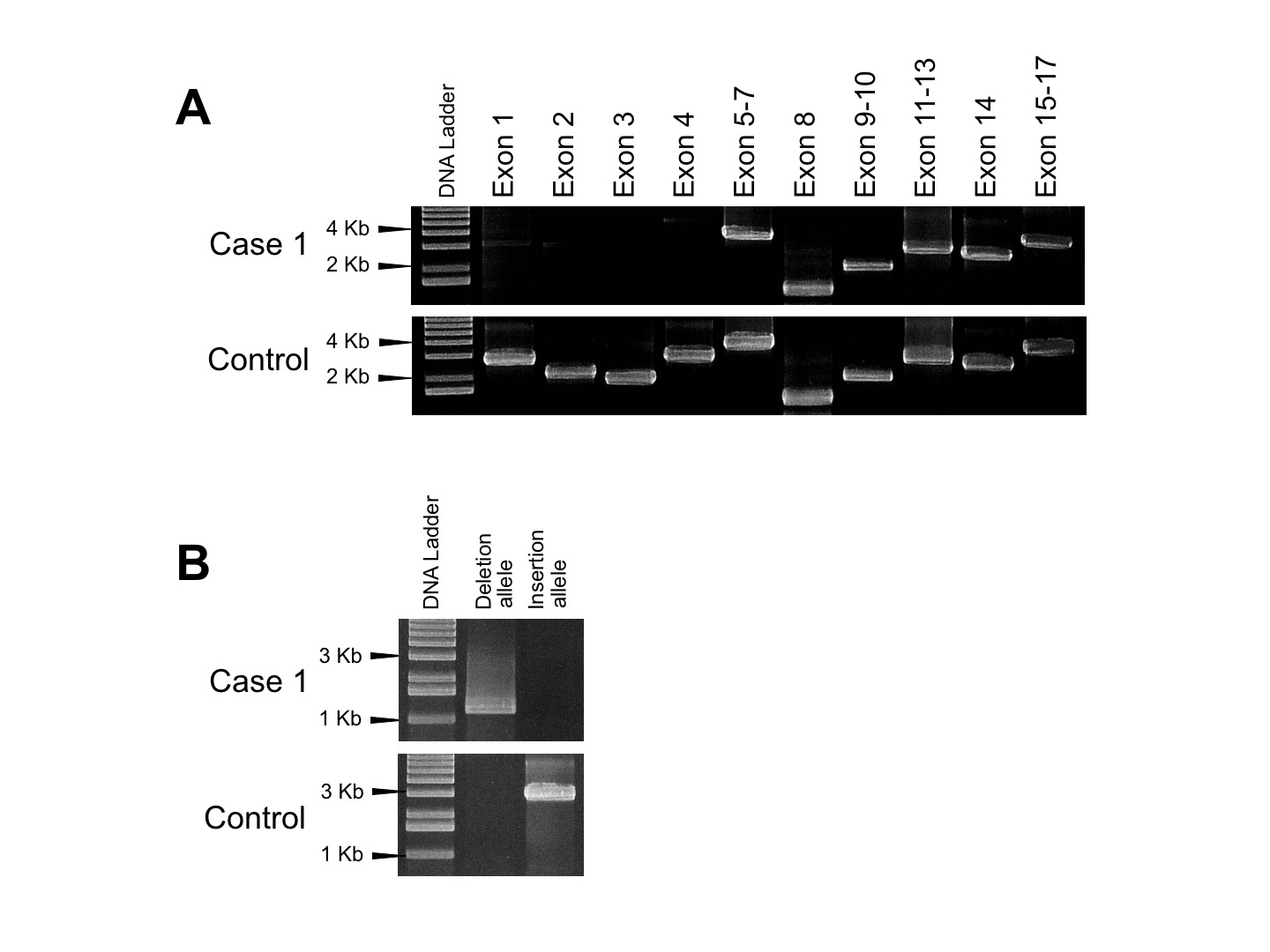
**

1. After the alignment of reads obtained from MiSeq sequencing to reference human genome, no read was mapped to exons 1 – 4 of *DRC1*, while all the other target exons were covered with appropriate depth of reads. Exons 1 – 17 of *DRC1* were re-amplified from genomic DNA of case 1 in 10 single PCR reactions using the primer pairs shown in **Table S1**, and the amplified products were electrophoresed in 1% agarose gel with GelRed (Biotium, CA, USA). Exons 1-4 of *DRC1* were not amplified by individual PCR, suggesting a large homozygous deletion in *DRC1*.
2. The DNA region encompassing the deletion was amplified only from the genomic DNA of case 1, while the insertion allele was amplified only from the control DNA.

**Figure S2**

***DRC1* deletion junction of case 2 in UNC**


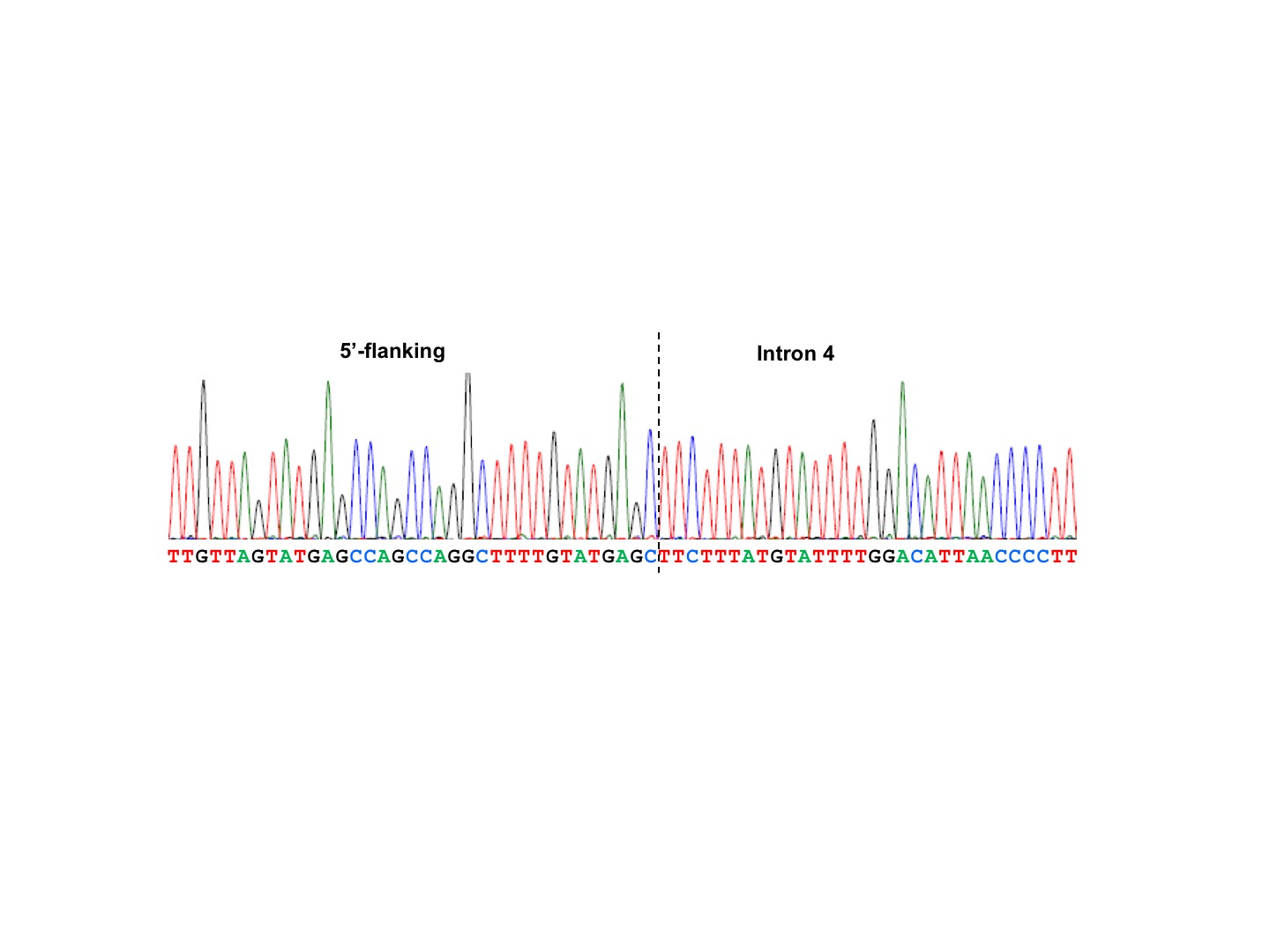


A genomic region containing the deletion was amplified by PCR and directly sequenced as described in SUPPLEMENTARY METHODS, and the deletion breakpoints were determined.

**Figure S3**

**Multiplexed PCR-based method to amplify deletion and insertion alleles**


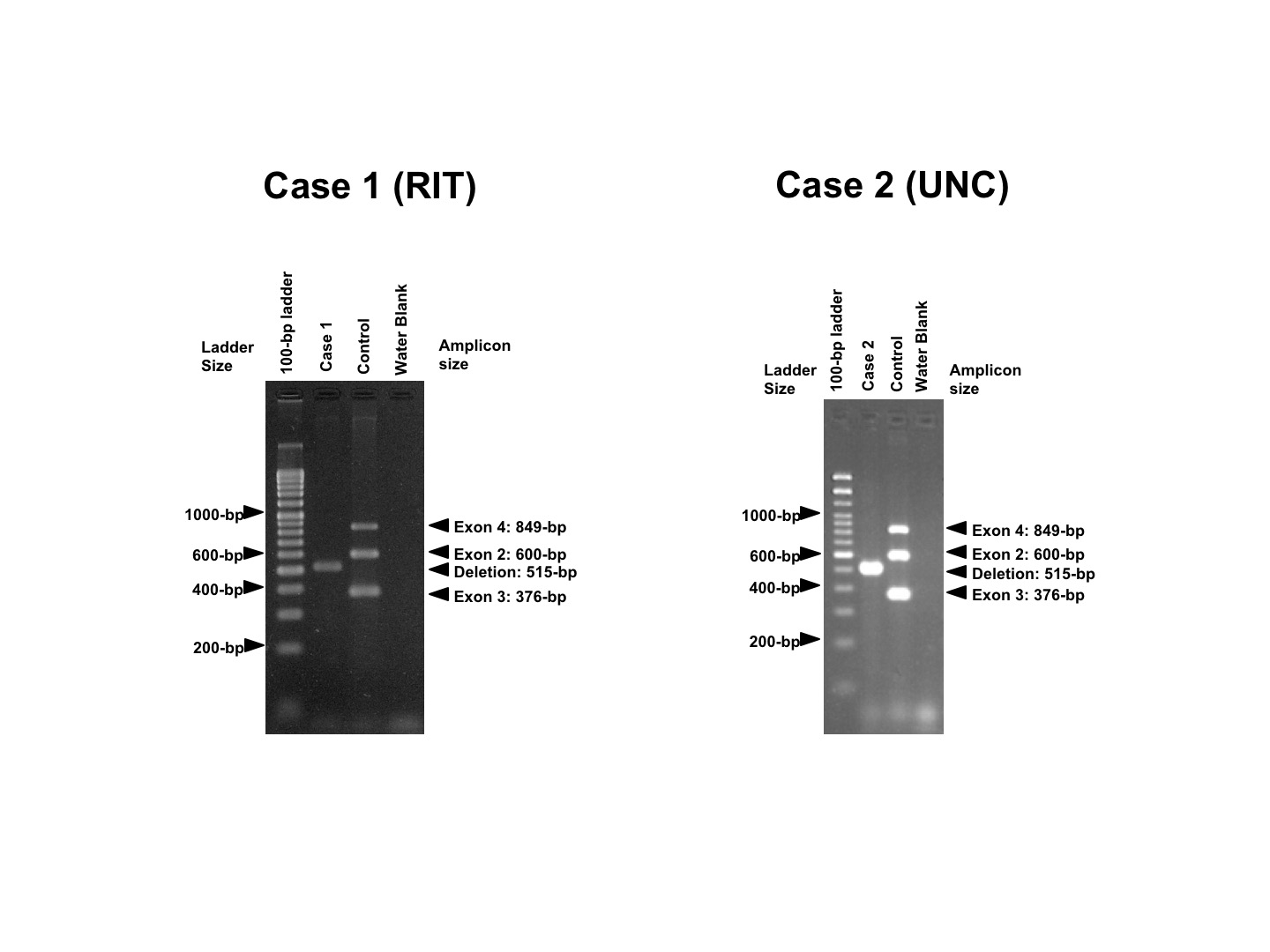


Genomic DNA from cases 1 and 2 were subjected to PCR to amplify deletion and insertion alleles at RIT and at UNC, respectively. The amplification of the single 515 bp fragment was observed in the cases, which indicated the homozygosity of the deletion allele, while the control genomic DNA gave three fragments (849 bp, 600 bp and 376 bp) from the insertion allele.
